# Biomarkers in preclinical cancer imaging

**DOI:** 10.1007/s00259-014-2980-7

**Published:** 2015-02-12

**Authors:** Monique R. Bernsen, Klazina Kooiman, Marcel Segbers, Fijs W. B. van Leeuwen, Marion de Jong

**Affiliations:** 1 Department of Nuclear Medicine, Erasmus MC Rotterdam, PO Box 2040, 3000 CA Rotterdam, The Netherlands; 2 Department of Radiology, Erasmus MC Rotterdam, Rotterdam, The Netherlands; 3 Department of Biomedical Engineering, Thorax Center, Erasmus MC Rotterdam, Rotterdam, The Netherlands; 4 Interventional Molecular Imaging Laboratory, Department of Radiology, Leiden University Medical Center, Leiden, The Netherlands

**Keywords:** Preclinical, Biomarker, Imaging, Molecular imaging, Cancer, Multimodality, Hallmarks

## Abstract

In view of the trend towards personalized treatment strategies for (cancer) patients, there is an increasing need to noninvasively determine individual patient characteristics. Such information enables physicians to administer to patients accurate therapy with appropriate timing. For the noninvasive visualization of disease-related features, imaging biomarkers are expected to play a crucial role. Next to the chemical development of imaging probes, this requires preclinical studies in animal tumour models. These studies provide proof-of-concept of imaging biomarkers and help determine the pharmacokinetics and target specificity of relevant imaging probes, features that provide the fundamentals for translation to the clinic. In this review we describe biological processes derived from the “hallmarks of cancer” that may serve as imaging biomarkers for diagnostic, prognostic and treatment response monitoring that are currently being studied in the preclinical setting. A number of these biomarkers are also being used for the initial preclinical assessment of new intervention strategies. Uniquely, noninvasive imaging approaches allow longitudinal assessment of changes in biological processes, providing information on the safety, pharmacokinetic profiles and target specificity of new drugs, and on the antitumour effectiveness of therapeutic interventions. Preclinical biomarker imaging can help guide translation to optimize clinical biomarker imaging and personalize (combination) therapies.

## Introduction

In connection with the increasing trend towards personalized medicine, the development of imaging biomarkers and quantitative imaging techniques has been identified as a major research priority in medical imaging communities [1–4]. Adhering to the definition of a biomarker proposed by the Biomarkers Definitions Working Group [5], an imaging biomarker is: “A characteristic that can be objectively measured from imaging data as an indicator of normal biological processes, pathogenic processes, or pharmacological responses to a therapeutic intervention”. In the clinical as well as the preclinical research setting, imaging biomarkers can be a measure of anatomical, physiological/functional or molecular characteristics (Table 1). Anatomical and functional imaging biomarkers, such as imaging-based tumour size measurements and tumour perfusion measurements, are routinely used in clinical studies, but are less commonly used in preclinical studies, and vice versa, the use of molecular imaging biomarkers is more common in preclinical studies. The latter often require the use of new chemical entities that require preclinical evaluation before they become safely applicable in humans [6].

**Table 1:** Examples of typical imaging biomarkers

Type	Characteristic	Imaging method	References
Anatomical	Tumour size/morphology	MRI, CT, US	[157]
Physiological/functional	Vessel density	CE MRI, CE CT, CE US	[205, 206]
	Vessel functionality	CE MRI, CE CT, CE US	[206–207]
	Cellular integrity	DW MRI	[185]
Molecular	Metabolic activity/metabolites	FDG PET/MRS	[209–210]
	Receptor expression	PET, SPECT, USMI, optical	[74, 118, 119, 125, 128, 130, 195]
	Enzymatic activity	PET, SPECT, MRI, optical	[18, 175, 177, 179]

*MRI* magnetic resonance imaging, *CT* computed tomography, *US* ultrasonography, *PET* positron emission tomography, *CE* contrast-enhanced, *DW* diffusion-weighted*, FDG* fluoro-d-glucose, *MRS* magnetic resonance spectroscopy, *USMI* ultrasound molecular imaging

Preclinical studies are very important to obtain more insight into and a better understanding of biological and pathological processes and to perform initial assessments of the therapeutic potential of newly developed drugs. Classically, such studies have been performed using large groups of animals and killing them at various time-points followed by histopathological examination of harvested tissue. With the current availability of high-resolution and highly sensitive preclinical imaging technologies many biological and pathological tissue characteristics can now be noninvasively and longitudinally assessed in living animals (Table 2). Not only does this allow reduced animal use, but it also provides more accurate information compared to the classical technologies [7, 8]. With the availability of animal imaging systems similar to clinical imaging systems, preclinical studies offer valuable options in providing proof-of-concept in the development process of new imaging biomarkers for clinical use.

**Table 2:** Overview of common in vivo small-animal imaging modalities

Technology	Means of detection	Resolution	Depth	Quantitative	Agents	Target	Relative cost
CT	Ionizing radiation (X-rays)	50 μm	No limit	Yes	Iodinated molecules	Anatomical, physiological	€€
PET	Ionizing radiation (γ-rays)	1 – 2 mm	No limit	Yes	^19^F-, ^64^Cu-, ^68^Ga-, or ^11^C-labelled compounds	Physiological, molecular	€€
SPECT	Ionizing radiation (γ-rays	0.3 – 1 mm	No limit	Yes	^99m^Tc-, ^111^In-, ^67^Ga-labelled compounds	Physiological, molecular	€€
MRI	Electromagnetism	10 – 100 μm	No limit	Yes	Paramagnetic and magnetic compounds (iron oxide; chelated Gd^3+^)	Anatomical, physiological	€€€
US	Acoustic waves	50 μm	Centimetres	Yes	Microbubbles	Anatomical	€
Optical	Light	1 – 5 mm	<3 cm	Yes	Luciferine, fluorochromes	Physiological, molecular	€

Adapted from: de Jong et al. [8]

Next to imaging systems, imaging agents are of crucial importance in biomedical imaging. Most commonly they are contrast agents and tracers that show accumulation at the target site after binding to receptor structures. Alternatively, specific enzymatic cleavage mechanisms may be exploited. Examples include: radiotracers, fluorescent molecules, paramagnetic ions or combinations thereof [9–18]. Small particles, including nanoparticles, liposomes and microbubbles, that can be (non)covalently bound to targeting molecules have also been developed [17, 19–23]. Such vectors are promising in the area of drug delivery and MRI, optical and photoacoustic imaging, contrast-enhanced ultrasonography and ultrasound molecular imaging, and thermoablative therapy [17, 19, 23–25]. Examples include ligand-functionalized polymer-shelled microcapsules [26] and mixed liposome/peptide/DNA (LPD) nanocomplexes [27] for nuclear and optical imaging as well as for MRI, illustrating the versatile potential of targeted and differentially labelled particles as research tools in cancer imaging.

**Fig. 1:**
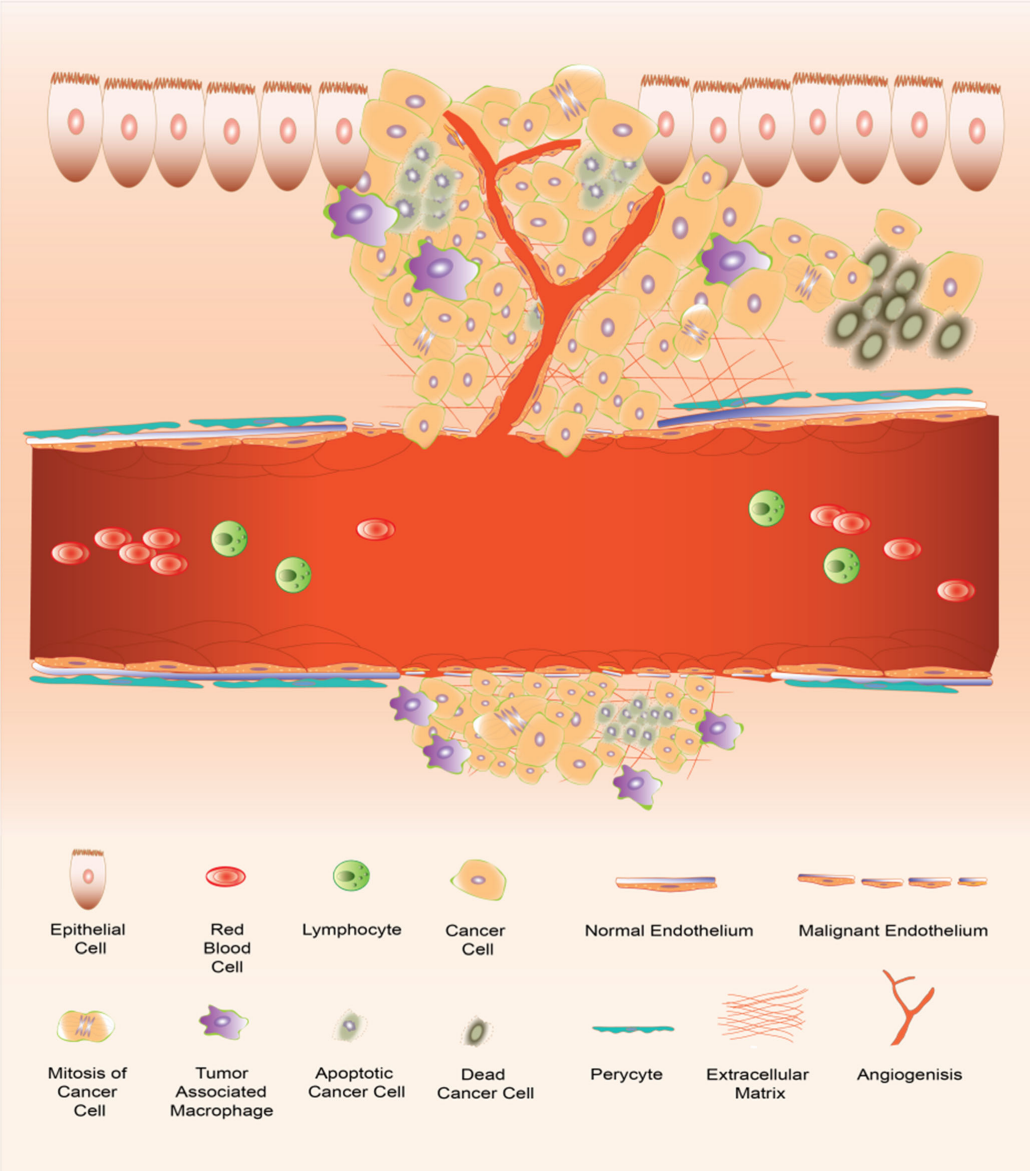
Hallmarks of cancer and imaging biomarkers (*1* uncontrolled proliferation, *2* angiogenesis, *3* altered metabolism, *4* invasion and metastasis, *5* inflammation and immune cells, *6* cell death)

In cancer research, the search for and use of imaging biomarkers has been strongly connected with the “hallmarks of cancer” defined in the past two decades (Fig. 1) [28, 29]. These hallmarks are considered crucial characteristics of tumours that define their level of malignancy and/or responsiveness for treatment. As such these characteristics can be considered indicative of a patient’s prognosis. Impressive developments in the areas of imaging technology and imaging tracers have strengthened preclinical imaging studies on the hallmarks of cancer. Following these hallmarks, in this review we describe the state of the art and future perspectives of imaging biomarkers in preclinical in vivo oncological studies, as well as recent successful translation into the clinic. We also address specific challenges encountered in preclinical research regarding the influence of animal handling techniques on research findings. Due to the focus on hallmarks and due to space constraints, we were not able to cover fully the extensive field of tumour imaging using tumour-specific markers, for example somatostatin receptors (SSTR), epidermal growth factor receptors (EGFR) and oestrogen receptors. Many of these markers are addressed in more detail in other, more clinically oriented, chapters of this special issue, and have also been discussed in various recent excellent reviews [30–33], to which the reader is referred to.

## Imaging biomarkers for the “hallmarks of cancer”

### Imaging proliferation/growth

Rapid and uncontrolled proliferation is the primary hallmark of cancer and underlies various other characteristics of tumours, e.g. angiogenesis and altered metabolic profiles [34]. High proliferative activity is often linked to tumour aggressiveness and is therefore considered a biomarker suitable for prognosis [35]. Furthermore, in radiotherapy-based treatment strategies the proliferative index of tumours is further considered a predictive biomarker of response. Reduction in proliferative activity in tumours, on the other hand, may function as a biomarker for therapeutic response assessment, especially with treatment strategies that have a primarily cytostatic effect. Consequently, significant effort has been dedicated to the development and validation of imaging biomarkers for tumour cell proliferation. Nuclear imaging techniques based on probing the so-called thymide salvage pathway, using tracers such as ^11^C-FMAU, ^18^F-FLT, and ^76^Br-BFU, are well-known examples for proliferation imaging [36]. In both preclinical [36] and clinical [35, 37] studies significant correlations have been found between uptake of these tracers and proliferative activity determined in tissue biopsies ex vivo. These tracers are still under investigation for their use as imaging biomarkers for early treatment response assessment. In various preclinical studies major decreases in ^18^F-FLT uptake were observed following antitumour treatment [38–41]. In these studies decrease in ^18^F-FLT uptake also coincided with reduced proliferative activity or reduced tumour growth. However, despite the fact that similar observations were made in clinical studies, various limitations of these techniques have also been identified, including incorporation into mitochondrial DNA instead of nuclear DNA and high uptake in liver and bone marrow. These characteristics limit the specificity of these tracers for actual tumour cell proliferation. For FLT, a specific limitation is the fact that ^18^F-FLT is not incorporated into DNA at all, but is only trapped in the cytosol [42]. FLT uptake is in that sense not directly related to DNA synthesis. This may be a main reason why in various forms of cancer no relationship between ^18^F-FLT uptake and proliferative index or response to treatment has been found [35, 43, 44]. Because of the encountered limitations of these thymidine analogues alternative approaches for cell proliferation imaging have been explored. Recent efforts in this respect include probing of type II topoisomerase (Topo-II) activity and expression levels of the sigma-2 receptor.

Topo-II is an ATP-dependent enzyme that is involved in cell cycle control and is essential in transcription, replication and chromosome segregation processes, and shows overexpression of one of its isoforms (Topo-IIα) in various types of cancer [45–47]. Next to being an attractive target for molecular therapy, Topo-II is therefore also considered an attractive target for imaging cell proliferation [45–48]. Some recent preclinical studies have shown the basic feasibility of generating imaging probes for Topo-IIα. Further development and optimization of these probes is required though, since the tracers generated to date show unfavourable biodistribution in vivo [48, 49].

**Fig. 2:**
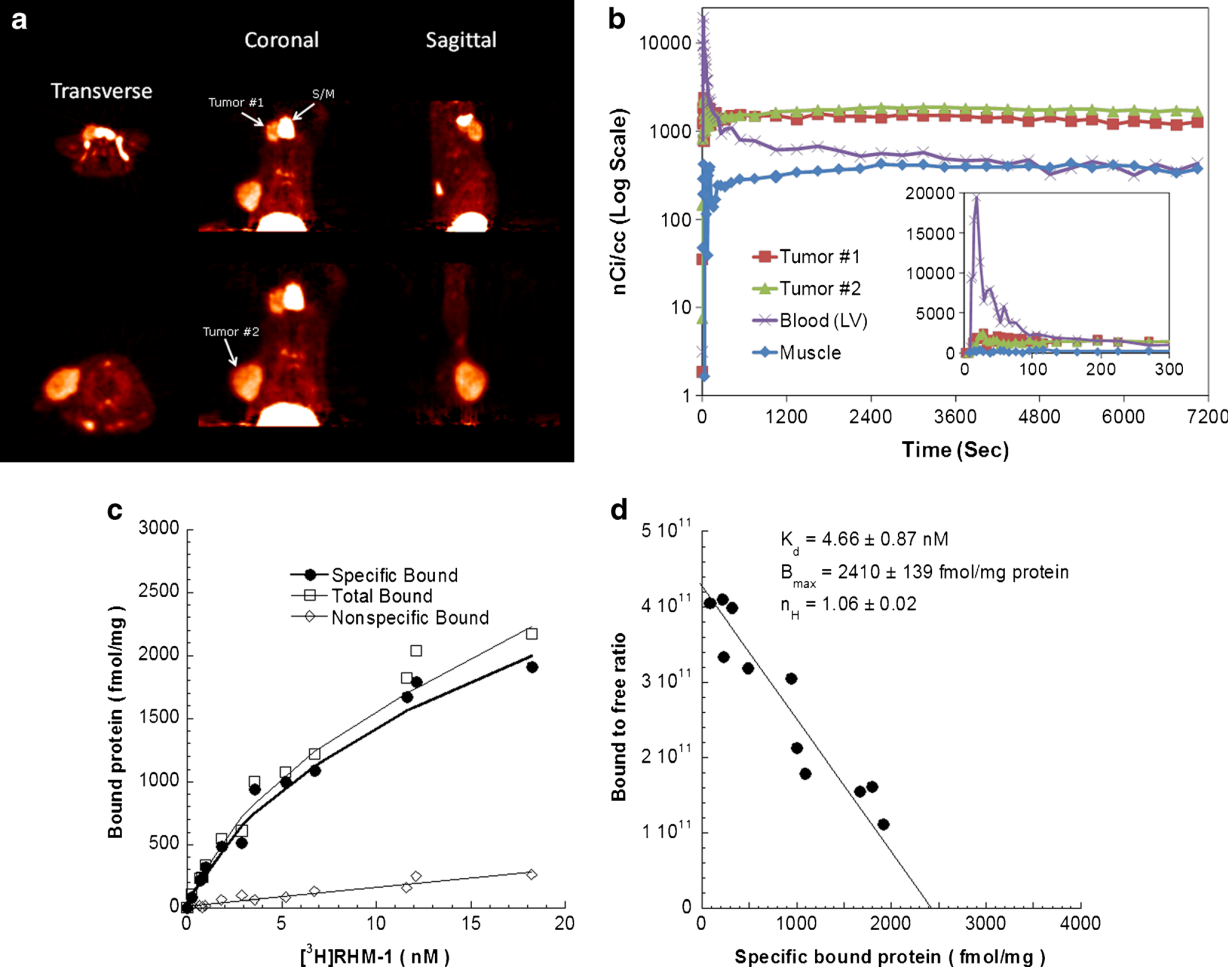
Characterization of the pharmacokinetics of [^18^F]ISO-1 and in vitro determination of sigma-2 receptor density in a rat model of mammary tumour induced by injection of *N*-methyl-*N*-nitrosourea. **a** Two-hour summed images show two tumours and the submandibular gland (*S/M*). The liver is evident in the coronal slices. **b** Time–activity curves of the two tumours, muscle and the left ventricular blood pool (*inset* shows the kinetics during the initial 5 min). **c** Representative saturation binding experiments which show the total bound, nonspecific bound and specific bound [^18^F]ISO-1. **d** Representative Scatchard plots which were used to determine the equilibrium dissociation constant (*K*_d_), the maximum number of binding sites (*B*_max_) and the Hill coefficient (*n*_H_). Reprinted from Shoghi et al. [57]

Another recently proposed target that may be suitable as an imaging marker of cell proliferation is the sigma-2 receptor. Sigma receptors are upregulated in rapidly proliferating cells, with the sigma-2 receptor being specifically overexpressed in proliferating tumour cells, i.e. tenfold compared to quiescent tumour cells [50]. Because of this specificity of sigma-2 receptor expression in actual proliferating tumour cells, it offers unique options in tumour imaging. Current efforts are therefore dedicated to the development of suitable imaging probes for the sigma-2 receptor [51–53]. A promising tracer in this respect is [^18^F]ISO-1. Uptake of this tracer has been shown to be significantly correlated with Ki-67 expression in animal models [54, 55], as well as in a first in-patient study [56]. In a recent preclinical imaging study, Shoghi et al. [57] evaluated the usefulness of this tracer in the measurement of the proliferative status of tumours and as a marker of early response. In two breast cancer xenograft models, they observed significant correlations between tumour uptake of the radiolabelled ligands and growth and proliferative status of the tumours (Fig. 2).

In conclusion, currently no validated imaging probe for the noninvasive assessment of the proliferative status of tumours exists. Recent preclinical studies have identified some potential relevant targets and generation and evaluation of probes specific for these targets are underway.

### Imaging tumour angiogenesis

Since angiogenesis is a critical process related to tumour growth and metastasis, a vast amount of effort has been put into the development and validation of imaging techniques for visualization and quantification of vessel density and vascular functionality, using (dynamic) contrast-enhanced imaging techniques. Angiogenesis is an imaging biomarker applied in tumour diagnosis and in the prediction and assessment of treatment response [58–60]. In recent years these techniques have also been increasingly used in the preclinical cancer research setting, including studies regarding the assessment of novel antiangiogenic treatment strategies, drug delivery studies and tumour model validation [61–63]. However, given the lack of standardization of these techniques and uncertainty in the interpretation of the derived parameters, which also holds true in the clinical setting, preclinical studies are also focused on defining the technological aspects of these techniques [64–66].

In the era of molecular medicine, molecular imaging approaches in general, including techniques for the assessment of angiogenesis and angiogenic processes, have been receiving increasing attention. So with the elucidation of molecular processes in angiogenesis, various imaging targets have been identified and are under investigation (Table 3). Often these involve membrane proteins expressed by endothelial cells, but proteins involved in angiogenesis and expressed by tumour cells or stromal cells are also targets of interest. Two of the most interesting targets in this respect are carbonic anhydrase IX [67, 68] and hypoxia-inducible factor-1 [69]. In many cases these targets are not only considered as imaging targets, but also as targets for treatment [70–72]. A variety of imaging methods, including PET, SPECT, MRI, ultrasonography and optical imaging, have been used in molecular imaging strategies.

**Fig. 3:**
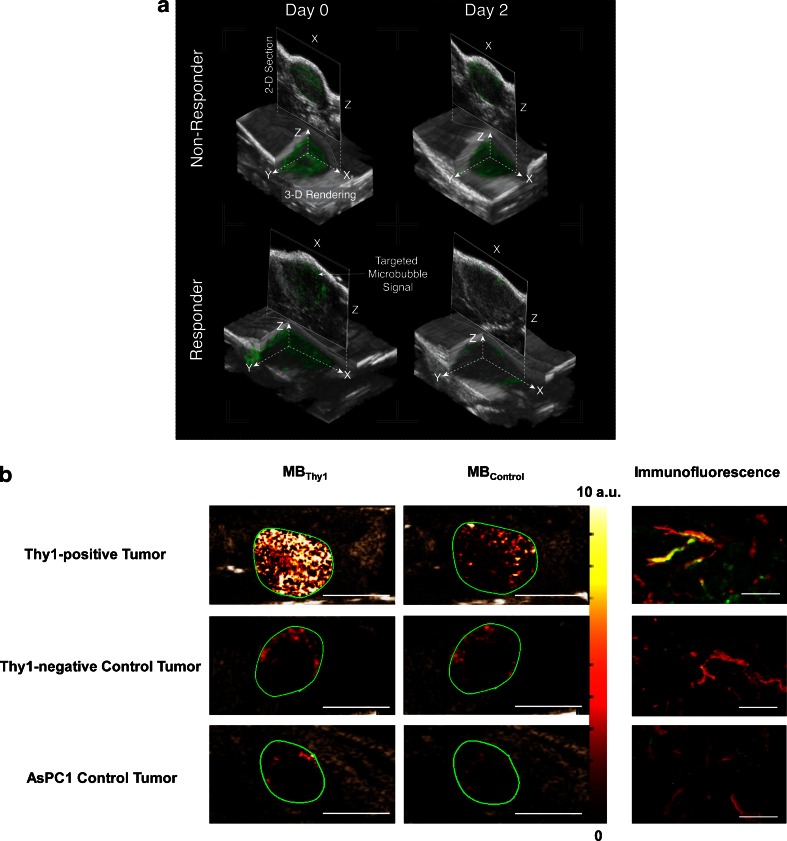
Ultrasound molecular imaging of tumour angiogenesis. **a** 3D images of a nonresponding (*top*) and a responding (*bottom*) pancreatic tumour in mice on day 0 and day 2 after aurora A kinase inhibitor treatment. The *green colour* represents the signal from the α_v_β_3_-targeted microbubbles which is overlain on the black and white B-mode ultrasound image. **b** The group of Willmann has overcome the problem of poor expression of human cancer-specific endothelial markers in murine models by developing a mouse model that expresses human vascular biomarkers. They transfected mouse endothelial cells with the human biomarker of interest and implanted these with the tumour cells of interest. Using this method, Foygel et al. [232] expressed human thymocyte differentiation antigen 1 (Thy 1 or CD90) in pancreatic ductal adenocarcinoma. *Left* The Thy1-targeted microbubble (MB_Thy1_) signal (colour-coded scale in arbitrary units) overlain on black and white B-mode ultrasound images is strong in Thy1-positive tumour, whilst there is only background signal in both types of control tumour. *Centre* There is also low signal from control-targeted microbubbles (MB_Control_; *green circles* tumour regions). *Right* Corresponding immunofluorescence micrographs (ex vivo) of merged double-stained sections (*red* murine CD31, *green* human Thy1), confirming human Thy1 expression on neovasculature in Thy1-positive tumours (*yellow*). *Scale bars*: 5 mm (*left* and *centre*), 50 μm (*right*). Reprinted from Tsurata et al. [233]

 A technique that has recently been gaining a lot of ground in these applications is ultrasound molecular imaging using targeted microbubbles (Fig. 3) [25, 73, 74]. In genetically modified mouse models, VEGFR2-targeted microbubbles have been shown to detect precancerous tissue, such as liver dysplasia [75] and breast hyperplasia. Ultrasound molecular imaging has also shown potential as an early response marker in several cancer types [76–79]. In recent studies the superiority of ultrasound molecular imaging over functional vascular imaging and tumour size measurements for response monitoring has been demonstrated in selected tumour models [80, 81]. This technique is now on its way to the clinic; a recent phase 0 clinical trial demonstrated that VEGFR2-targeted microbubbles can be successfully used for prostate cancer imaging [82] and to have the potential for monitoring patients at high risk of cancer, such as aggressive primary hepatocellular carcinoma [83]. In the preclinical setting new developments in this field involve the use of 3D imaging techniques and microbubbles functionalized with ligands specific for tumour-specific markers (Fig. 3).

**Table 3:** Selected examples of molecular targets as imaging biomarkers of angiogenesis

Target	Preclinical/Clinical use	References
Integrins	Clinical, preclinical	[73, 212–219]
VEGFR2	Clinical, preclinical	[76, 220–222]
VEGF	Preclinical	[223, 224]
Tumour endothelial marker 8 (TEM8)	Preclinical	[225, 226]
CD147	Preclinical	[227–229]
CD276	Preclinical	[230]
EGFR	Preclinical	[227, 231]

Additional molecular imaging strategies regarding the use of imaging biomarkers for the assessment of tumour angiogenesis involve imaging approaches to assess tissue hypoxia, a physiological effect strongly linked to the aberrant tissue vasculature in many tumours [84]. Tumour oxygenation can be imaged by optical techniques [85, 86], MRI [67, 87–89], photoacoustic techniques [90] and nuclear techniques [91, 92]. These approaches are in most cases based on the detection of haemoglobin saturation using techniques such as BOLD MRI, or accumulation of tracers following a reduction reaction by which the reduced molecule becomes entrapped or bound within tumour cells or tissue, for example with nitroimidazole-based probes.

In view of current antiangiogenic treatment strategies molecular imaging of angiogenic processes is very much in focus. At the preclinical level various targets have been identified and are currently being evaluated. A number of these have also entered clinical testing, but data obtained so far have not resulted in consistent and conclusive results, and more studies are warranted.

### Imaging cellular energetics

Rapid cell growth and hypoxic conditions are considered driving forces behind altered energy metabolism, as is often found in tumours. However, oncogenetically driven processes have also been described as underlying causes of altered energy metabolism. In tumours, specifically the high degree of reliance on glucose as the metabolic substrate and the so-called Warburg effect provide a basis for metabolism imaging. The Warburg effect entails the phenomenon of aerobic glycolysis, where even under normoxic conditions tumour cells convert glucose into lactate. The preferred use of glucose as substrate in many tumour types is the reason for the avid uptake of fluorodeoxyglucose (FDG) and the reason for the high clinical value of ^18^F-FDG in cancer diagnostics and treatment response monitoring. The use of FDG as imaging biomarker for tumour localization, prognosis and response has been addressed in various excellent clinical reviews to which the reader is referred to [93–95].

 Because some limitations in the use of ^18^F-FDG PET have also been recognized, e.g. accumulation in non-tumour tissue and limited uptake in slow-growing tumours such as prostate cancer and neuroendocrine tumours, alternative methods for metabolic profiling are also under investigation. Since tumours may also display changes in protein and phospholipid metabolism, these processes also provide imaging targets [96–98]. New insights into the role of amino acids and amino acid transporters have instigated the development and evaluation of new radiolabelled amino acids, as recently reviewed by Huang and McConathy [96]. Findings of altered phospholipid metabolism have resulted in the development and testing of radiolabelled choline analogues [99, 100]. Schwarzenbock et al. [101] recently addressed the issue of sensitivity and specificity of three such tracers in a xenograft prostate cancer model in mice. They found that the new tracer [^11^C]SMC performs better than the clinically used [^11^C]CHO. Emonds et al. on the other hand reported a potential limitation of choline-based tracers [102]. Comparing [^11^C]CHO and [^11^C]acetate in androgen-dependent and androgen-independent prostate cancer xenograft models, they found that androgen deprivation influences the uptake of [^11^C]CHO, and warned of the risk of underestimation of the presence of recurrent prostate cancer following androgen deprivation therapy.

**Fig. 4:**
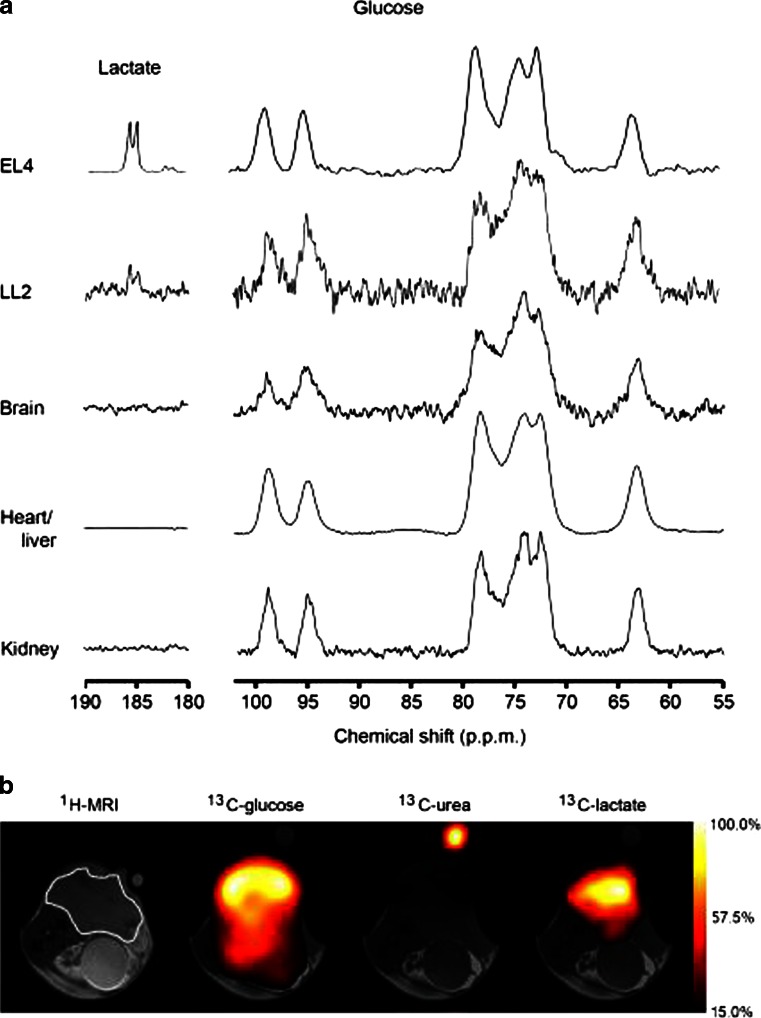
^13^C spectroscopic imaging showing the spatial distribution of labelled glucose and lactate in EL4 and LL2 tumour-bearing mice. **a** Representative ^13^C MR spectra acquired from subcutaneous EL4 and LL2 tumours, brain, heart, liver and kidneys 15 s after injection of 0.35 mL 100 mM hyperpolarized [U-^2^H, U-^13^C]glucose. The lactate spectra are the sum of four transients collected over 1 s, whereas a single transient was acquired for the glucose spectra. Flux of the hyperpolarized ^13^C label was only observed between [U-^2^H, U-^13^C]glucose (63 – 99 ppm) and lactate C1 (doublet at about 185 ppm) in EL4 and LL2 tumours. **b** Representative chemical shift selective images obtained about 15 s after intravenous injection of 0.4 mL 200 mM hyperpolarized [U-^2^H, U-^13^C]glucose into an EL4 tumour-bearing mouse. The spatial distribution of glucose, urea and lactate are shown as voxel intensities relative to their respective maxima. The ^1^H MR images, shown in grey scale, were used to define the anatomical location of the tumour (*outlined in white*). A urea phantom was included to serve as a reference. The colour scales represent arbitrary linearly distributed intensities for the hyperpolarized images. Reprinted from Rodrigues et al. [112]

Magnetic resonance spectroscopy (MRS) has played a major role in metabolic profiling of tumours for several decades [103]. However, due to issues regarding sensitivity and the need for specialized techniques, MRS has not yet evolved as a routine clinical practice. Nonetheless, MRS approaches continue to be explored for certain types of cancer [104] with recent increasing interest in its use in prostate cancer [105, 106] and breast cancer [107, 108]. The interest in MRS-based assessment of tumour metabolism has recently undergone a further boost by the newly developed technique of hyperpolarized MRI [109]. This technique is based on MRS imaging of ^13^C-labelled cell substrates that have undergone dynamic nuclear polarization (or hyperpolarization). The hyperpolarization step has the big advantage of enhancing the sensitivity of detection of ^13^C-labelled compounds by more than 10,000-fold [110], by which one of the main limitations of ^13^C-MRS can be overcome, thus allowing sensitive assessment of the dynamics of metabolic processes in vivo. This technique has opened up new possibilities in studying metabolic pathways in tumours by which a better understanding of the sometimes controversial metabolic signatures in tumours can be obtained [111]. Also, the potential use of this technique for response assessment in cancer therapy was recently demonstrated. Rodrigues et al. found highly tumour-specific conversion of hyperpolarized [U-^2^H, U-^13^C]glucose to lactate and a marked decrease in the lactate/glucose ratio 24 h after treatment with the chemotherapeutic drug etoposide in murine tumour models of lymphoma and lung tumours (Fig. 4) [112]. In prostate cancer cell lines, Canapè et al. [113] demonstrated the ability of hyperpolarized NMR, using [5-^13^C]glutamine as a probe, to noninvasively assess glutaminolysis. They were also able to show that the rate of glutaminolysis in prostate tumour cell lines changed depending on their survival response after treatment with cytostatic drugs, and therefore argued that hyperpolarized [5-^13^C]glutamine metabolism is a promising biomarker for the noninvasive detection of tumour response to treatment.

In conclusion, imaging of the metabolic profile of tumours has already been part of clinical routine in cancer management. Current techniques, however, still have various limitations regarding sensitivity, specificity or applicability, and additional and alternative methods are needed. Various promising new techniques are under evaluation and multimodal imaging approaches may solve some the issues encountered [114].

### Imaging tumour evasion and metastasis

One of the most critical factors in clinical cancer management is the degree of tumour evasion and the occurrence of (distant) metastasis. These features determine if local or systemic therapies are required and may be the basis for palliative rather than curative therapy. Routine molecular imaging technology using ^18^F-FDG (molecular marker: increased sugar metabolism) has revolutionized the noninvasive detection of tumour extent and metastatic spread for a great number of cancers (see above). Unfortunately, this highly generic approach is not always effective, meaning that some cancer types require more dedicated techniques. One example is the sentinel node procedure that is focused on the identification of (submillimetre) micrometastasis in the lymphatic track using lymphatic flow and accumulation by the immune system as molecular markers [115]. Early studies have shown that more personalized means to monitor the extent of oncological disease may benefit greatly from the use of molecular tumour markers [116–118]. As indicated in the Introduction, many clinical success stories in molecular imaging of specific cancer biomarkers can be found in which tumour-specific markers, such as SSTR-targeting peptides and HER-2 (EGFR2)-targeting antibodies, have been used [119–121]

A number of molecular targets are considered to be representative of metastatic disease and have provided the basis for the development and recent translation of innovative imaging agents. These targets include prostate-specific membrane antigen (PSMA), chemokine receptor 4 (CXCR4) and mesenchymal–epithelial transition factor (c-MET). PSMA is expressed both on the primary tumour and on prostate cancer-related metastasis, so it has the potential to visualize the full tumour load, including metastasis [122]. PSMA-based imaging agents described are based on molecules that differ in size, e.g. antibodies, nanobodies, aptamers and low molecular weight inhibitors of PSMA [116, 123–127]. The last of these in particular have shown high potential in mice and more recently also in humans [116]. Important to note here is that clinical PSMA PET is so effective that it might rapidly replace choline-based PET [128]. Although clinical trials with optical derivatives have not yet been reported, it has already become clear from preclinical studies that these small molecules remain efficient when a fluorescent dye is attached [129].

Based on the potential to drive migration along a stromal derived factor 1 (SDF-1) gradient, CXCR4 is considered a marker of malignant/metastatic disease. High expression levels of SDF-1 have been found at the most common sites of cancer metastasis, e.g. lymph nodes, lungs, liver and bone marrow. This makes CXCR4 a candidate target for molecular imaging to define the extent of disease and/or to identify highly aggressive subpopulations of tumour cells [130, 131]. Preclinical molecular imaging of CXCR4 is focused around SPECT, PET and optical imaging. Where the first two have shown potential for the noninvasive visualization of disease extent [132, 133], the last enables microscopic evaluation of receptor interactions and has demonstrated potential in image-guided surgery applications [134]. Studies regarding this receptor nicely illustrate that the efforts to optimize affinity and kinetics have paid off. Of all the new compounds tested in the preclinical setting, to our knowledge only one has made it to use in humans, namely [^68^Ga]pentixafor [135]

MET (a receptor tyrosine kinase) is an oncogene that plays a role in tumour metastasis and motility [136]. Tyrosine kinase MET is the receptor for hepatocyte growth factor (HGF/SF) and interaction between MET HGF/SF can induce scattering and migration of (tumour) progenitor cells. The general occurrence on carcinomas makes MET a marker for metastatic risk stratification. MET imaging has been pursued using different targeting moieties and different imaging labels. For example, anticalins and antibodies have been used to generate PET tracers for this receptor [137, 138]. Both show activity in vitro and in mouse tumour models. Alternatively, peptides have been used to optically visualize MET in mice. Examples are the linear peptide cMBP-AOC-Cy5.5 [139] and the cyclic peptide GE137 (that also contains a Cy5-like dye) [140]. To our knowledge only the latter has so far found its way into clinical trials, where it has shown potential for fluorescence-guided surgery of colorectal neoplasia.

In conclusion, the recent successful development of tracers for PSMA, CXCR and c-MET indicates the value that exploratory preclinical studies have in the field of molecular imaging. Given the many ongoing preclinical imaging efforts, it is highly likely that more tracers for metastatic disease will find their way into the clinic. When this is the case, it is of course critical that they are evaluated beyond exploratory first-in-human studies. Ultimately, (randomized) multicentre studies will be required to prove the clinical value of the new technologies.

### Imaging inflammation and (evasion of) immune cells

Tumours harbour dynamic microenvironments in which cancer cells are associated with normal host cells. The tumour-associated stroma plays an important role during tumour growth, influencing events such as angiogenesis, metastasis and immune suppression [28, 141, 142]. As such, the stroma forms an attractive target for diagnostic and therapeutic applications. To distinguish normal from cancer cells, different strategies can be followed. Mice and other animal models can be created that use genetic reporters to label or track specific cells within the tumour or cells can be labelled with tumour-targeting or high-affinity molecules that contain radionuclides, fluorochromes or magnetic labels. Different myeloid cells are important components of the tumour stroma. Myeloid cells are frequently found to infiltrate tumours and have been linked to diverse tumour-promoting activities. In particular, tumour-associated macrophages (TAMs) are an important component of the tumour stroma [143]. Macrophages are plastic cells that can adopt different phenotypes depending on the immune context; microenvironmental stimuli can drive a macrophage either towards a “classical” (M1) or an “alternative” (M2) activation state, two extremes in a spectrum. M1 macrophages are typically characterized by the expression of proinflammatory cytokines, inducible nitric oxide synthase 2 and MHC class II molecules. M2 macrophages have a decreased level of these molecules, are identified by a malignant phenotype and their signature expression of a variety of markers, including arginase-1 and mannose and several receptors. Strongly proangiogenic TAMs that reside in hypoxic tumour areas express high levels of macrophage mannose receptor (CD206) [144, 145].

It has been suggested that TAMs display an M2-like phenotype [144]. However, because of a lack of specific imaging agents, there is a poor understanding of their absolute numbers, flux rates and functional states in different tissues. Molecular probes for macrophage imaging target several aspects of macrophage cell biology. Cellular probes specific for membrane markers on the cell surface can localize macrophages within tissues, and surface proteins whose levels increase in stimulated cells can preferentially identify activated cells. Surface targets for macrophage imaging, although not specific for this cell type, include vascular cell adhesion protein-1 [146], receptors (folate receptor, SSTR subtype 2) [147, 148], intercellular adhesion molecule-1 [149], and chemokine receptors [150]. In addition to localization by targeting surface proteins, internalization of probes through phagocytosis by macrophages can also detect such cells preferentially; several nanoparticle-based and superparamagnetic probes show promise in this regard [151].

Cell-based therapies, such as immunotherapy and stem cell therapy, are most promising anticancer therapies; many forms of adoptive T cell therapy are on the verge of being translated to the clinic [43, 152, 153]. The development of therapeutic strategies using tumour-targeted cells requires the ability to image and determine in vivo the location, distribution and viability of the therapeutic cells, as well as their biological fate with respect to cell activation and differentiation. Such cell-tracking methods, including labelling with, for example, [^111^In]oxine, or magnetofluorescent techniques for cell labelling, play an important role in basic cancer research, where they serve to elucidate novel biological mechanisms [154–156].

In conclusion, it is expected that the material described, which allows visualization of the biology of macrophages and other immune cells in vivo in preclinical models, will also be useful for a multitude of human applications. Because of the implications of stromal cells and factors, this is an emerging field of potential targets for both imaging and therapy.

### Imaging cell death

The currently most widely used therapy response criteria are based on size measurements of tumour lesions according to RECIST or WHO criteria [157]. However, lesion size changes after therapy may take a long time to occur, and lesion size may not always be reflective of actual response, i.e. eradication of tumour cells. Lesion size measurements are therefore considered not to be ideal for early response assessment, which is often desired in drug efficacy trials and treatment monitoring. This has led to a high level of interest in noninvasive methods for assessing tumour cell death following interventions, allowing early therapy response assessment [158].

Cell death is characterized by loss of cellular integrity that is mediated by a large variety of molecular changes including: externalization of phosphatidylserine to the outer leaflet of the plasma membrane bilayer, activation of effector caspases, depolarization of the plasma and mitochondrial membranes and loss of plasma membrane integrity [159, 160]. All these processes have been studied as targets for imaging biomarkers with the presentation of phosphatidylserine residues at the outer surface of the plasma membrane being the most widely studied to date [158, 161]. For this target, annexin-V-based probes have been most frequently used, including probes suitable for imaging by MRI [162, 163], PET [164], SPECT [165], optical techniques [166, 167] and ultrasound molecular imaging [168]. However, despite promising results in clinical trials [169, 170], suboptimal biodistribution profiles of annexin-V tracers [171] have stimulated the search for other molecules that can bind to phosphatidylserine [172–174].

 As well as phosphatidylserine exposure, detection of caspase activity has also been investigated as an imaging biomarker of cell death [175–178] and has even reached testing in early clinical trials [179]. Besides these two main molecular targets for cell death imaging, several other approaches are also being evaluated as (surrogate) markers of cell death. These involve imaging probes or imaging techniques that are able to visualize membrane depolarization or loss of membrane integrity. Regarding depolarization of membranes, triphenyl phosphonium-based probes [68, 180, 181], and 2-(5-fluoropentyl)-2-methyl malonic acid [182–184] have been found to show uptake characteristics in tumour tissue that could be linked to tumour cell death and reductions in tumour volume as verified by other imaging techniques or ex vivo analyses of tumour tissue.

Interest in two MRI techniques has recently been increasing as a means to image processes related to tumour cell death: diffusion-weighted (DW) MRI [185] and MRS of hyperpolarized fumarate [186]. DW MRI provides image contrast through measurement of the diffusion properties of water within tissues. By using sequential imaging with different weightings for diffusion an apparent diffusion coefficient (ADC) map can be generated. Water diffusion is restricted within cells and increases following loss of cellular integrity by which ADC DW imaging may be used to monitor cell death. Various in vivo studies have shown significant correlations between increases in ADC values in tumour tissue and response to treatment and apoptosis of tumour cells [187–190]. Due to loss of cellular integrity during cell death, fumarate can enter cells rapidly and is converted to malate. This process can be monitored by MRS of hyperpolarized [1,4-^13^C_2_]fumarate [186]. The sensitivity of this latter technique has even been shown to be superior to that of ADC DW imaging [189].

In conclusion, since eradication of tumour cells is the ultimate goal of anticancer therapy, cell death detection is considered of high importance for (early) response assessment. Several methods and probes are currently under investigation with questions still remaining regarding the choice of relevant imaging target and the timing of assessment [1, 158, 191].

## Multifunctional probes

Here we briefly introduce some concepts regarding multifunctional imaging probes such as theranostic and multimodality probes. As indicated above many of the molecular targets mentioned are also suitable as targets for targeted therapy. This has formed the basis for theranostics (theragnostics), the principle by which the same targeting molecule or particle can be used for both diagnosis and targeted therapy. This principle has been exploited in peptide receptor radionuclide therapy [9], but is also being considered a valuable strategy in the development and use of other therapeutics, e.g. biologicals and particle-based drug delivery systems such as liposomes, microcapsules and polymeric micelles [192, 193].

Multimodality probes consist of compounds that carry multiple signalling beacons by which they can be imaged by two or more imaging modalities [194, 195]. These compounds have the advantage that the strengths of different modalities can be combined, e.g. high sensitivity and high spatial resolution or quantitative performance, and may specifically play a role in image-guided drug delivery [193] and image-guided surgery [196].

## Specific challenges in preclinical imaging studies

Preclinical studies in animal models are important for the development and evaluation of new imaging techniques and imaging probes. However, data obtained in in vivo molecular imaging studies in small animals may be influenced by the animal model used, by animal preparation and handling, and by the use of anaesthesia. Therefore, we briefly address some important issues to be taken into account during small-animal imaging in general (more detailed information can be obtained from the literature [197]).

The use of multimodality imaging may be very demanding for an animal, mostly because, in contrast to human studies, imaging of small animals generally requires anaesthesia. It is important to note that this may confound the results of imaging studies, as anaesthesia may influence many physiological parameters [198]. Such issues especially need to be taken into account when multimodality imaging studies are performed at regular time intervals. The feasibility of such studies is strongly dependent on parameters such as the total acquisition time, the type of anaesthesia administered, the surgical procedures required per imaging session and the body temperature of the anaesthetized animal, which depends on the use of a heated bed before and during scanning. Imaging conscious animals or imaging animals after death may avoid the issues with anaesthesia, but these approaches clearly have their own inherent disadvantages and problems. For ultrasound imaging, the type of carrier gas for isoflurane anaesthesia affects the longevity of the microbubbles. Longevity of nontargeted microbubbles is longer when medical air is used instead of oxygen [199–201]; this might also have implications for targeted microbubbles. On the other hand, longer persistence of freely floating targeted microbubbles would also prolong the ultrasound molecular imaging protocol as typically imaging is not performed until most freely floating targeted microbubbles are cleared from the circulation [73, 74]. However, new developments aiming at distinguishing adherent from freely floating targeted microbubbles form an active area of research.

Besides anaesthesia issues, ionizing radiation used in imaging studies can cause side effects and undesired antitumour effects. In small-animal imaging, often relatively high amounts of radioactivity have to be administered to produce high-resolution images within a reasonable acquisition time. In small-animal SPECT imaging, on a body weight basis, the activity dose is therefore up to 100 times higher than in the clinical setting. Funk et al. [202] estimated that the whole-body dose in preclinical SPECT and PET studies ranges between 6 and 90 cGy in mice and between about 1 and 27 cGy in rats. They concluded that the whole-body dose in small-animal imaging can be very high in comparison to the lethal dose to mice. The dose should therefore be monitored carefully and the administered activity should be kept to a minimum. In follow-up SPECT/CT studies, the risk of side effects due to high radiation doses from consecutive scanning must be considered and dosimetry should be performed, and should also include the radiation dose delivered by (repeat) CT scans performed in SPECT/CT [203]. Willekens et al. [204] estimated that the median organ dose in mice from a standard micro-CT scan is about 40 cGy and this may influence the experimental outcome, but adaptation of the scan protocol allows accurate imaging without the risk of interfering with the experimental outcome of the study. Note that high-resolution (high-dose) scanning can always be planned as a one-off scan or as the final CT scan of a longer nuclear scan series.

## Concluding remarks

We have described a panel of biological targets derived from the “hallmarks of cancer” and indicated their potential for use as imaging biomarkers in oncology based on data obtained from preclinical studies in animal tumour models. Such preclinical studies are crucial in providing proof-of-concept in the development process of new imaging biomarkers for diagnostic, prognostic or early response monitoring purposes in patients. Furthermore, the use of imaging biomarkers in the preclinical setting is also of value in the evaluation of new drugs and tracers. It allows high-throughput assessment of basic safety, pharmacokinetics and target specificity of the compound of interest before clinical testing. While proof-of-concept has been provided for some of the described imaging biomarkers, the majority still need further validation at both the preclinical and clinical level before they can qualify as robust imaging biomarkers.

 Therefore, driven by medical need the search for new or improved imaging targets, imaging probes and (multimodal) imaging technologies will continue. Multimodality imaging is a promising new area in this respect. Recent imaging advances are synergistic with new imaging agents, reporters, better models and labelling options, so finally it is hoped that molecular imaging systems will allow clinicians to routinely ‘see’ expression and activity of specific molecules, cells and/or biological processes influencing tumour behaviour, that will answer important questions to ultimately offer cancer patients treatment tailored to their individual characteristics.
